# Supplementary material for: The genome-wide mutational consequences of DNA hypomethylation
Source: Sci Rep. 2023 Apr 27;13:6874. doi: 10.1038/s41598-023-33932-3 (PMC10140063; doi:10.1038/s41598-023-33932-3)
Supplement: Supplementary file 1 — Supplementary Information. [file 41598_2023_33932_MOESM1_ESM.pdf]

Supplementary information to:

The genome-wide mutational consequences of DNA hypomethylation

Nicolle Besselink[1,§], Janneke Keijer[1,§], Carlo Vermeulen[1], Sander Boymans[1], Jeroen de Ridder[1], Arne van Hoeck[1], Edwin Cuppen[1,2], Ewart Kuijk[1,3,4*]

[1]Center for Molecular Medicine and Oncode Institute, University Medical Center Utrecht, Utrecht, The Netherlands.

[2]Hartwig Medical Foundation, Amsterdam, The Netherlands

[3]Division of Pediatric Gastroenterology, Wilhelmina Children's Hospital, University Medical Center Utrecht, Utrecht, The Netherlands.

[4]Regenerative Medicine Center Utrecht, Uppsalalaan 8, 3584 CT, Utrecht, The Netherlands.

[§]Contributed equally

[*]Corresponding author: E.W.Kuijk-3@umcutrecht.nl

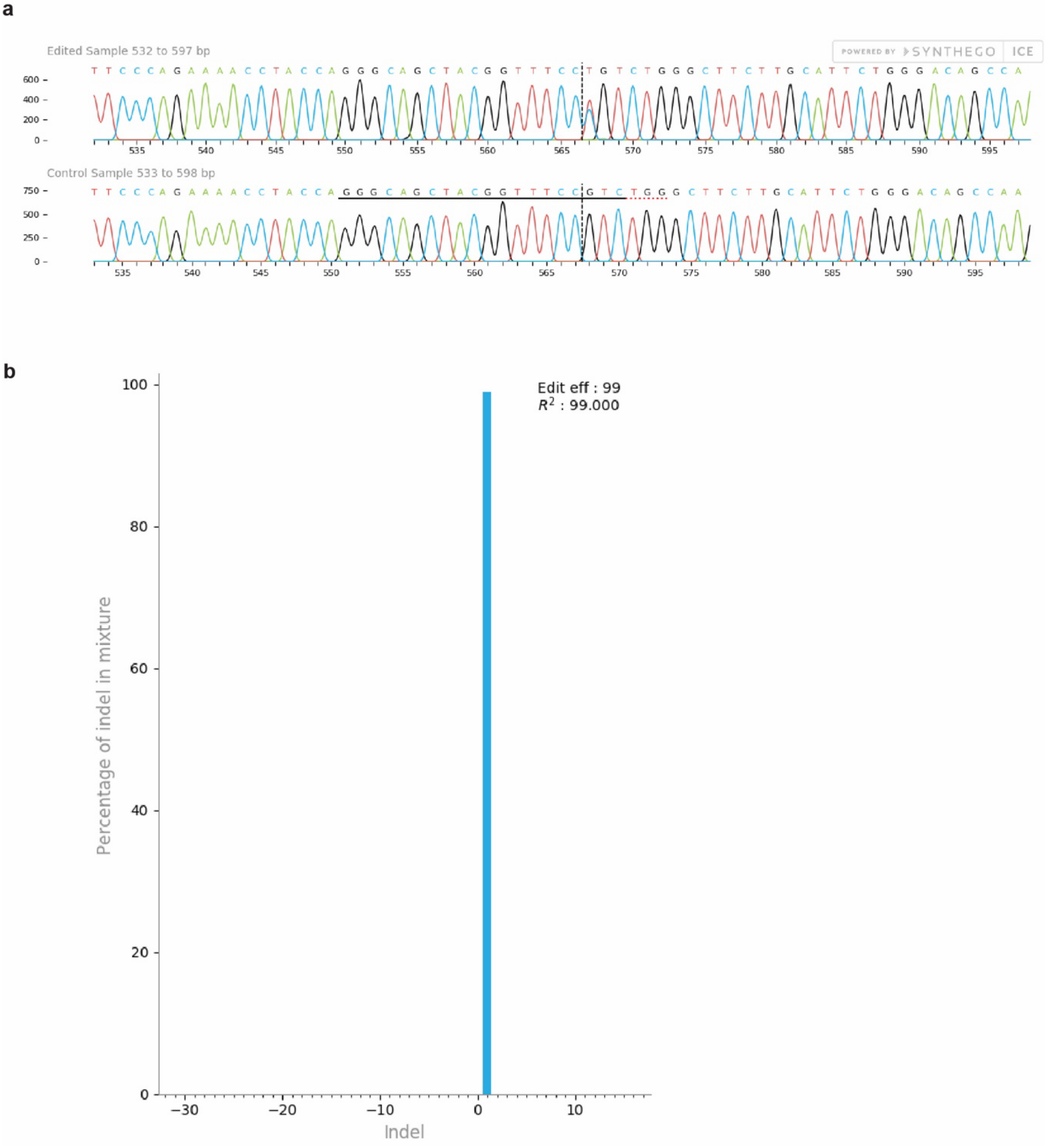

Supplementary Figure 1: Validation of TP53 knockout

(a) Sanger sequencing result of PCR amplified region in TP53 from RPE-1 cells targeted by CRISPR/CAS9 (top panel) and control cells (bottom panel). Line indicates the sgRNA sequence that was used for targeting. (b) CRISPR edits were inferred from the Sanger trace with the ICE CRISPR Analysis Tool from Synthego [69].

a

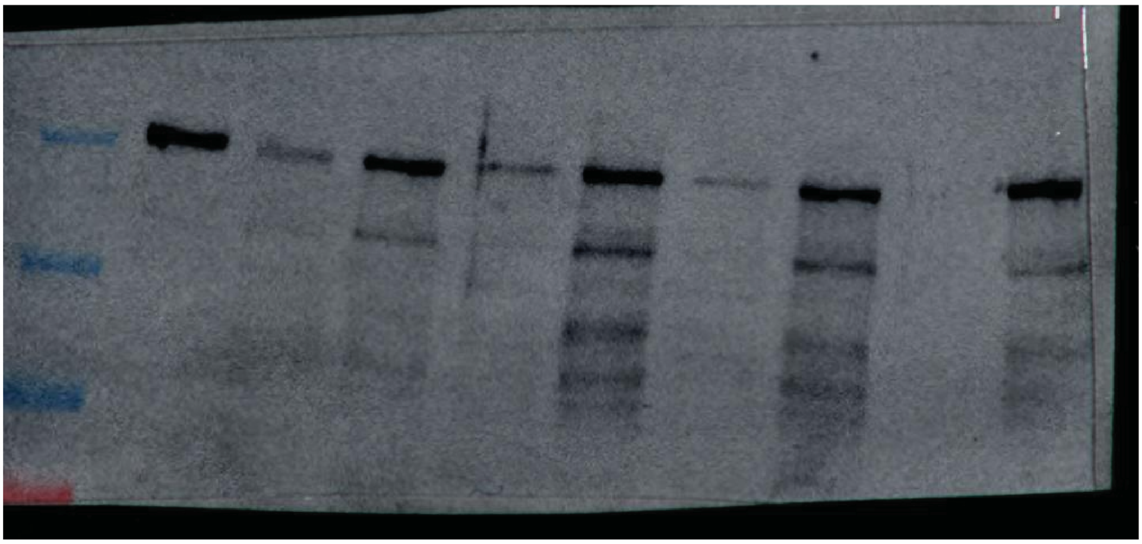

b

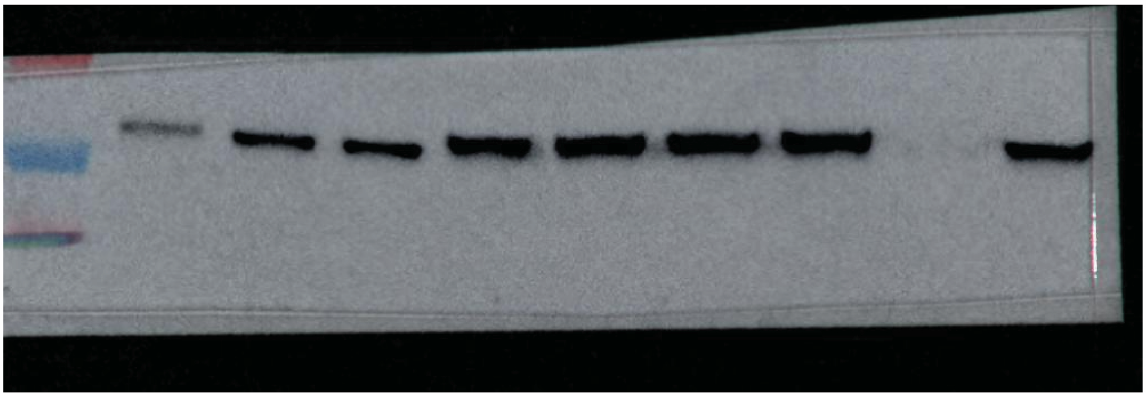

Supplementary Figure 2: Full length original western blots for which cropped versions are shown in Figure 1c. (a) Uncropped image for DNMT1. (b) Uncropped image for Tubulin. For both panels: lane 1 is protein ladder, lane 2 are untreated cells at start of the experiment, lanes 3-8 are *DNMT1kd* knockdown cells treated with or without doxycyline for 1 week (lanes 3+4), 2 weeks (lanes 5+6), 3 weeks (lanes 7+8), and 4 weeks (lanes 9+10).
